# Unraveling Desmoid-Type Fibromatosis-Specific Health-Related Quality of Life: Who Is at Risk for Poor Outcomes

**DOI:** 10.3390/cancers14122979

**Published:** 2022-06-16

**Authors:** Anne-Rose W. Schut, Emma Lidington, Milea J. M. Timbergen, Eugenie Younger, Winette T. A. van der Graaf, Winan J. van Houdt, Johannes J. Bonenkamp, Robin L. Jones, Dirk. J. Grünhagen, Stefan Sleijfer, Cornelis Verhoef, Spyridon Gennatas, Olga Husson

**Affiliations:** 1 Department of Medical Oncology, Erasmus MC Cancer Institute, 3015 GD Rotterdam, The Netherlands; a.schut@erasmusmc.nl (A.-R.W.S.); m.timbergen@erasmusmc.nl (M.J.M.T.); w.vd.graaf@nki.nl (W.T.A.v.d.G.); s.sleijfer@erasmusmc.nl (S.S.); 2 Department of Surgical Oncology, Erasmus MC Cancer Institute, 3015 GD Rotterdam, The Netherlands; d.grunhagen@erasmusmc.nl (D.J.G.); c.verhoef@erasmusmc.nl (C.V.); 3 Sarcoma Unit, Royal Marsden NHS Foundation Trust, London SW3 6JJ, UK; emma.lidington@kcl.ac.uk (E.L.); eugenie.younger@doctors.org.uk (E.Y.); robin.jones4@nhs.net (R.L.J.); spyridon.gennatas@nhs.net (S.G.); 4 Department of Medical Oncology, Netherlands Cancer Institute, 1066 CX Amsterdam, The Netherlands; 5 Department of Surgical Oncology, Netherlands Cancer Institute, 1066 CX Amsterdam, The Netherlands; w.v.houdt@nki.nl; 6 Department of Surgical Oncology, Radboud University Medical Center, 6525 GA Nijmegen, The Netherlands; han.bonenkamp@radboudumc.nl; 7 Division of Clinical Studies, Institute of Cancer Research, Royal Marsden NHS Foundation Trust, London SM2 5NG, UK; 8 Department of Medical Oncology, Guy’s and St Thomas’ NHS Foundation Trust, London SE1 9RT, UK

**Keywords:** desmoid-type fibromatosis, rare diseases, health-related quality of life, patient-reported outcomes, disease-specific measures

## Abstract

**Simple Summary:**

Desmoid-type fibromatosis (DTF) is an uncommon, non-metastasising soft-tissue tumour. Patients can experience a wide variety of disease-specific issues related to the unpredictable clinical course and aggressiveness of DTF, negatively impacting their health-related quality of life (HRQoL). Little is known about which DTF patients are particularly affected by an impaired HRQoL. In the current study, HRQoL was evaluated among different groups of DTF patients, using the EORTC QLQ-C30 and the DTF-QoL, a DTF-specific HRQoL questionnaire. Age, sex, presence of comorbidities, and type of treatment were found to be most strongly associated with DTF-specific HRQoL outcomes. In general, socio-demographic factors had the greatest impact on generic HRQoL, whereas the influence of clinical factors was mainly seen on the DTF-QoL, underlining the importance of a disease-specific questionnaire. Knowledge of the differences in DTF-specific HRQoL between subgroups can be used to individualize the HRQoL-measurement strategy for research and clinical practice.

**Abstract:**

Desmoid-type fibromatosis (DTF) is a rare, soft-tissue tumour. These tumours do not metastasize, but their local aggressive tumour growth and unpredictable behaviour can have a significant impact on health-related quality of life (HRQoL). Little is known about which DTF patients are particularly affected by an impaired HRQoL. The objectives of this study were to assess HRQoL among different groups of DTF patients and to investigate which socio-demographic and clinical characteristics were associated with DTF-specific HRQoL. A cross-sectional study was conducted among DTF patients from the United Kingdom and the Netherlands. HRQoL was assessed using the European Organization for Research and Treatment of Cancer Quality of Life Questionnaire Core 30 (EORTC QLQ-C30), accompanied by the DTF-QoL to assess DTF-specific HRQoL. The scores were compared amongst subgroups, based on the socio-demographic and clinical characteristics of DTF patients. Multiple linear regression analyses with a backward elimination were conducted to identify the factors associated with DTF-specific HRQoL. A total of 235 DTF patients completed the questionnaires. Female patients, patients with more than two comorbidities, or patients who received treatment other than only active surveillance (AS) or surgery scored significantly worse on the subscales of both the EORTC QLQ-C30 and DTF-QoL. Patients that were ≥ 40 years scored significantly worse on the physical functioning scale of the EORTC QLQ-C30, while younger patients (18–39 years) scored significantly worse on several DTF-QoL subscales. Differences in the DTF-QoL subscales were found for tumour location, time since diagnosis and the presence of recurrent disease. Furthermore, treatments other than AS or surgery only, female sex, younger age and the presence of comorbidities were most frequently associated with worse scores on the DTF-QoL subscales. This study showed that (DTF-specific) HRQoL differs between groups of DTF patients. Awareness of these HRQoL differences could help to provide better, personalised care that is tailored to the needs of a specific subgroup.

## 1. Introduction

Desmoid-type fibromatosis (DTF) is a rare, intermediate-grade, soft-tissue tumour [1]. The estimated incidence in the population is 5–6 patients per million people per year. It usually affects young adult patients and tumours can be located in nearly any part of the body, most commonly, in the extremities and abdominal wall [2,3,4]. DTF does not metastasize, but it can display locally aggressive tumour growth, causing significant morbidity [1]. The biological behaviour of DTF is unpredictable and variable, and includes phases of progressive growth or growth stabilisation and spontaneous regression in 28% of tumours [5,6,7]. Regardless of the tumour’s behaviour or size, patients may experience a variety of symptoms, from no symptoms at all to extreme pain or functional limitations.

The most recent global consensus guideline recommends active surveillance (AS) as a frontline approach for asymptomatic and mildly symptomatic patients, independent of the tumour’s location or size [8]. After initial AS, the majority of DTF patients do not need active treatment, minimising overtreatment and potential treatment-related morbidity [7,9]. In the case of radiological or clinically significant progression or increasing symptoms, active treatment, including systemic therapies, surgical resection and local therapies, such as radiotherapy, can be considered [8]. With high local recurrence rates for DTF at anatomic sites other than the abdominal wall and treatment-related toxicities, these interventions do not guarantee tumour reduction or clinical benefit [3,8,10,11]. For a substantial proportion of patients, DTF is a chronic condition and the primary goal in treating DTF patients is to maintain an acceptable health-related quality of life (HRQoL) [12,13].

HRQoL is a multidimensional concept that includes the patient’s perception of the impact of their disease and treatment on their physical, psychological, and social functioning [14]. There are a limited number of studies focusing on HRQoL in DTF patients. These studies have shown that the diagnosis of DTF, its treatment, or both can have a significant impact on different domains of their HRQoL. From qualitative interview studies, it is known that DTF patients experience a variety of disease-specific issues associated with the rarity of DTF, the unpredictable clinical course and the variable treatment efficacies. Additionally, DTF patients report pain and physical symptoms caused by the tumour itself, or as a side effect of treatment [13,15,16]. These DTF-specific HRQoL issues are not captured by generic or cancer-generic HRQoL questionnaires, such as the European Organization for Research and Treatment of Cancer Quality of Life Questionnaire Core 30 (EORTC QLQ-C30), which are predominantly used in DTF studies and in clinical care [17]. Therefore, a DTF-specific HRQoL questionnaire, the DTF-QoL, was recently developed by our group, which can be used to evaluate the prevalence of HRQoL issues in DTF patients [18,19]. Furthermore, the small number of previous studies focused on the population of DTF patients as a whole because of small sample sizes. Consequently, little is known about the differences between subgroups of DTF patients, for example, about the differences in HRQoL between patients receiving different types of treatment or with tumours in different anatomic locations. The objectives of this study are to evaluate the HRQoL in different groups of DTF patients using the DTF-QoL and the EORTC QLQ-C30, and to investigate which socio-demographic and clinical characteristics are associated with DTF-specific HRQoL. The results of this study will provide important insights into the problems and needs of specific groups of DTF patients, which will help to identify patients at risk of a poor HRQoL and to better provide personalised care.

## 2. Materials and Methods

### 2.1. Study Sample and Data Collection

The sample included DTF patients from the United Kingdom (UK) and the Netherlands (NL), who participated in the QUALIFIED study (The evaluation of health-related quality of life issues experienced by patients with desmoid-type fibromatosis, registered at clinicaltrials.gov (accessed on 12 May 2022): NCT04289077) [18]. The QUALIFIED study is an international, multicentre, cross-sectional, observational study among adult (≥18 years) patients with sporadic DTF who were treated in one of the participating centres (one centre in the UK, three centres in the NL). After obtaining their informed consent, the patients completed a set of questionnaires, including the EORTC QLQ-C30 and DTF-QoL. Questionnaire data were collected via the PROFILES management system—an established international registry for the collection of cancer patient-reported outcomes [20]. Ethical and institutional approval was obtained in each participating centre in the UK and the NL. Further details of the protocol are described elsewhere [18].

### 2.2. Study Measures

#### 2.2.1. Socio-Demographic and Clinical Characteristics

Socio-demographic and clinical data were extracted from the questionnaire (patient-reported) and from the patient medical records. The questionnaire included single items on age, sex, race, marital status, family composition, educational level, employment status, tumour location, details regarding the diagnosis, received treatments and tumour recurrence. Comorbidities were assessed using an adapted self-administered comorbidity questionnaire (SCQ) [21], which included one question about the presence of comorbidities in the previous twelve months. Additional medical data were obtained from the electronic patient records to ensure correct and detailed reporting [18]. To compare the HRQoL between the different types of treatment, DTF patients were assigned to one of the following three treatment groups: “only AS”, “only surgery” and “other treatment”. Receiving treatment with non-steroidal anti-inflammatory drugs (NSAIDs) or other analgesics was not considered an active treatment [8]. The other treatment group included patients who received systemic therapy (i.e., chemotherapy, hormonal therapy, targeted medical therapy), local therapy (i.e., radiotherapy, isolated limb perfusion, high-intensity-focused ultrasound, cryoablation) or a combination of any form of active treatments. In addition, patients who received “only systemic therapy”, “only local therapy” or “combination of active treatments” were assessed as separate groups.

#### 2.2.2. Questionnaires

The EORTC QLQ-C30 was used to measure HRQoL [17]. This 30-item HRQoL questionnaire consists of five functional scales, a global quality of life scale, three symptom scales and a number of single items that assess common symptoms and the perceived financial impact of the disease. The timeframe of the questions is during the past week. Each item is scored on a Likert scale ranging from 1, “not at all” to 4, “very much”, with the exception of the global QoL scale, which is scored on a seven-point response scale ranging from 1, “very poor” to 7 “excellent”. Scores of all scales and single items are linearly transformed to a score between 0 and 100, according to the guidelines of the EORTC quality of life group [22]. A higher score on the functional scales and global quality of life means better functioning and HRQoL, whereas a higher score on the symptom scales means a higher symptom burden.

The DTF-specific HRQoL was measured by the DTF-QoL [19]. The DTF-QoL was developed according to the guidelines of the EORTC Quality of Life Group to supplement the EORTC QLQ-C30 and to assess the disease-specific issues that DTF patients experience [19,23]. The questionnaire consists of 96 items, which are divided into 3 symptom scales, 11 disease impact scales, and 6 single items. The timeframe of the symptom scales is the past week; the disease impact scales and single items have a timeframe of since diagnosis, except for the question on sexual interest, which has a timeframe of four weeks. Items are scored on a Likert scale, with a range of 1, “not at all” to 4 “very much”, with an additional “not applicable” option for certain questions. Scores of the DTF-QoL scales are calculated according to the EORTC QLQ-C30 scoring manual for symptom scales/items [22]. First, a raw score is obtained by estimating the average of the items that contribute to a scale. After a linear transformation of the raw scores of all scales and single items, scores range from 0 to 100. A higher score indicates a higher level of symptoms or problems.

### 2.3. Statistical Analyses

Patient characteristics were summarised using descriptive statistics. Continuous variables were presented as a mean and standard deviation (SD) or median and interquartile range (IQR) where skewed. The categorical variables were described as numbers and percentages. The differences in mean scores of the DTF-QoL and EORTC QLQ-C30 subscales between the subgroups of DTF patients were analysed using the Mann–Whitney *U* test in the case of two groups. In the case of more than two groups, an analysis of variance (ANOVA) with post hoc Bonferroni analysis was used. The clinically relevant differences in DTF-QoL scores between the treatment groups were determined with Norman’s “rule of thumb”, using the value of 0.5 SD as the default value for a clinically relevant difference [24]. A series of multiple linear regression analyses were conducted to investigate the association between clinical (comorbidity, time since diagnosis, treatment received, recurrence and tumour location) and socio-demographic characteristics (age, sex, relationship status, education level and current employment status) and the DTF-QoL scores. The categorical variables education level, comorbidity, treatment received and tumour anatomic location, had >2 categories and were transformed into dummy variables, with, respectively, low, none, only AS and abdominal wall as the reference groups. A manual backward elimination method was applied to determine the inclusion of variables in the final model, whereby, only those variables with a *p* < 0.05 were retained [25]. If any of the dummy variables had a *p*-value of < 0.05, the entire categorical variable was retained. If one of the dummy variables had the largest *p*-value and none of the dummy variables had a *p*-value of < 0.05, the entire categorical variable was eliminated. Given the large number of subscales, we decided not to give an extensive description in the text of the differences in scale scores and between which groups these differences were observed, but to refer to the tables as much as possible instead. All analyses were performed using SPSS software, version 25.0 (SPSS Inc., Chicago, IL, USA) and the figures were generated with GraphPad Prism, version 5.0 (GraphPad Software, La Jolla, CA, USA). For all analyses, *p*-values of <0.05 were considered statistically significant.

## 3. Results

### 3.1. Patient Characteristics

Two hundred and thirty-five DTF patients completed the DTF-QoL and EORTC QLQ-C30 questionnaires (response rate 46%). No statistically significant differences in sex, age at the time of diagnosis, and age at the time of the questionnaire were observed between the responders and non-responders. The socio-demographic and clinical characteristics of the study sample are described in Table 1. Most patients were female (*n* = 173, 73.6%) with a median age of 39.3 years (IQR 31.4–50.6) at the time of diagnosis. The median time since diagnosis for all patients was 4.7 years (IQR 2.3–7.8). The most common tumour locations were the abdominal wall (*n* = 58, 24.7%) and trunk (*n* = 54, 23.0%). Eighty-seven patients (37.0%) were treated with AS only and 64 patients (27.2%) with surgery only. The other active treatment types are specified in Table S1. Sixteen patients (6.8%) were undergoing active treatment at the time they completed the questionnaire. Back pain (*n* = 46, 19.6%), depression/anxiety (*n* = 41, 17.4%), joint condition (*n* = 26, 11.1%) and high blood pressure (*n* = 26, 11.1%) were the most common self-reported comorbidities.

### 3.2. Comparison of DTF-Specific HRQoL between Different Groups of DTF Patients

The mean HRQoL scores for the total sample and all subgroups of DTF patients on the DTF-QoL subscales and single items are presented in Table 2 and Table S2. Several differences were found for socio-demographic factors. Younger patients (18–39 years) experienced significantly more problems in six subscales, with the largest difference in the subscale parenting and fertility, previously described as the “parents and fertility” subscale. Female patients had significantly higher scores, indicating more problems, on four subscales. Unemployed patients experienced more problems in three subscales, with the highest score on the impact scale related to job and education.

Significant differences in the subscales of the DTF-QoL were also seen for clinical factors (Table 2). Having multiple comorbidities resulted in significantly worse scores on eight subscales. A longer time since diagnosis (≥5 years) resulted in significantly higher scores on eight subscales. Patients with recurrent disease experienced more problems in six subscales. Compared to tumours in some other anatomic locations, patients with tumours in the upper and lower extremities, hip/pelvis/gluteal region, and head and neck, scored significantly worse on several subscales. The lower extremity and hip/pelvis/gluteal group experienced significantly more symptoms that were related to physical consequences. Patients with tumours in the upper extremities or hip/pelvis/gluteal region scored higher on pain and discomfort. Tumours in the head and neck region resulted in more problems with employment and education.

**Figure 1:**
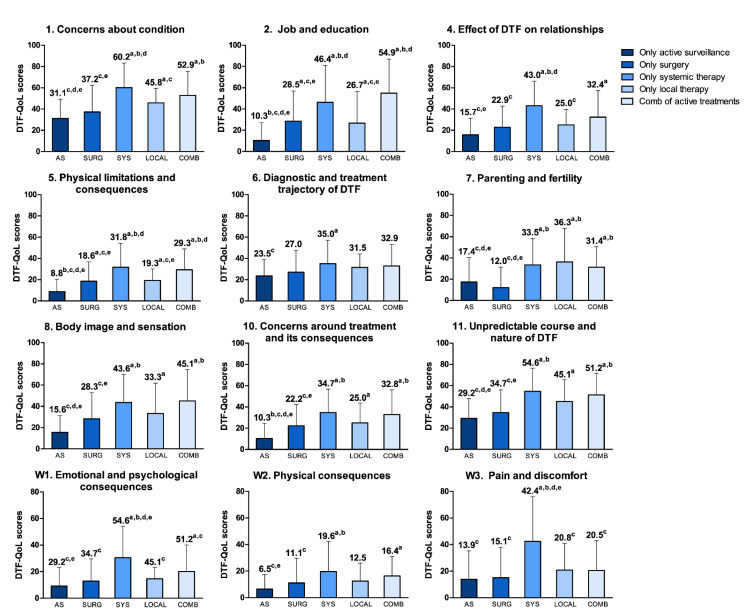
Mean DTF-QoL scores per treatment type. Differences in mean scores of DTF-QoL scales between treatment groups. Higher scores indicate a higher level of symptomatology/problems. Scale 3 (doctor-patient relationship, communication and information) and 9 (supportive care) are not shown because no significant differences were found between the treatment groups for these scales. Active surveillance, surgery, systemic therapy or local therapy only: including patients who received analgesics. Systemic therapy includes: chemotherapy, hormonal therapy and targeted medical therapy (tyrosine kinase and gamma-secretase inhibitors). Local therapy includes: radiotherapy, isolated limb perfusion, high-intensity-focused ultrasound, cryoablation. Combination of active treatments: including patients who received different combinations of surgery, systemic therapy or local therapy. ^a,b,c,d,e^ Corresponds to whether the score of the respective treatment group is clinically relevant different (difference ≥ 0.5 SD) compared to: ^a^ only active surveillance, ^b^ only surgery, ^c^ only systemic therapy, ^d^ only local therapy, ^e^ combination of active treatments. Abbreviations: AS, only active surveillance; Surg, only surgery; Sys, only systemic therapy; Comb, combination of active treatments.

With the exception of the subscales doctor-patient relationship and supportive care, and the single item wasting the time of cancer specialists, significant differences between the three treatment groups were seen for all DTF-QoL subscales and single items, with the other treatment group scoring higher than the group of patients who received AS or surgery only (Table 2 and Table S2). Figure 1 presents the mean DTF-QoL scores per treatment type and the clinically relevant differences between the treatment groups, considering systemic therapy and local therapy as separate groups.

### 3.3. Comparison of HRQoL between Different Groups of DTF Patients

The mean HRQoL scores for the EORTC QLQ-C30 are presented in Table 3 for the total sample and all the subgroups of DTF patients. Patients that were ≥ 40 years scored significantly lower on physical functioning and had significantly more problems with dyspnoea and sleep. Female patients had significantly worse scores on six subscales. Unemployed patients scored significantly lower on all functioning scales and on global health and had higher scores on the single items fatigue, dyspnoea, sleep and financial difficulties. Having multiple comorbidities resulted in lower scores on all subscales. No differences were seen in the time since diagnosis. There were significant differences between the three treatment groups in physical, role, emotional and social functioning, in global health and in fatigue, pain, sleep, diarrhoea and financial difficulties symptom items and scales. For most of these scales and symptoms, patients who received other treatments experienced more problems or symptoms than those patients receiving AS or surgery only. The presence of recurrent disease resulted in significantly worse scores in two subscales. Patients with tumours located in the hip/pelvis/gluteal/ region and the lower and upper extremities scored significantly higher on the pain items.

### 3.4. Factors Associated with DTF-Specific HRQoL

Multiple linear regression analyses with backward elimination were conducted to identify the socio-demographic and clinical characteristics associated with DTF-specific HRQoL (Table 4). An older age (≥40 years) was negatively associated with physical symptoms, while a younger age (18–39 years) was negatively associated with the impact of DTF on concerns about condition, relationships, parenting and fertility, body image concerns about treatment and its consequences, and the unpredictable disease course. Female sex was associated with more physical symptoms and problems related to job and education, physical limitations, parenting and fertility, and body image. Having one or more comorbidities was negatively associated with all the subscales, except for job and education, diagnostic and treatment trajectory, and parenting and fertility. Time since diagnosis was associated with only two scales, with fewer years since diagnosis being negatively associated with pain and discomfort, and a longer diagnosis with problems related to supportive care. Treatment other than AS or surgery only was associated with more problems on all DTF-QoL subscales, except for doctor-patient relationship and supportive care.

## 4. Discussion

This international, cross-sectional study evaluating HRQoL in DTF patients, showed that both generic and disease-specific HRQoL differ between subgroups based on socio-demographic and clinical characteristics of DTF patients. In multivariate analyses, younger age, female sex, presence of comorbidities, and treatment other than AS or surgery only, were most strongly associated with worse DTF-specific HRQoL outcomes.

The type of treatment a patient received was found to be one of the most important factors associated with both the generic and DTF-specific HRQoL. The group of patients who received systemic therapy or a combination of active treatments scored significantly worse than patients who received AS or surgery alone, with the differences in the HRQoL scores being clinically relevant. These results may be explained by the fact that patients who require systematic therapy or multiple treatments are those with more complicated DTF tumours, with a more aggressive disease course and/or in whom an eventual resection would be mutilating. The greater impact of these types of treatment may therefore be partly caused by a higher tumour burden. The variable response to systemic and local therapies in DTF may exacerbate the differences between those who need active treatment and those who do not. The treatment itself, or its side effects, could also affect HRQoL. For example, DTF patients undergoing systemic therapy reported comparable hair and skin problems to soft-tissue sarcoma patients who received chemotherapy, which can have a negative impact on the patient’s self-image [26]. In addition, a failure of (multiple) treatments can lead to uncertainties about the disease and treatment efficacy [13,15,16]. In general, HRQoL outcomes of patients who received AS or surgery only were comparable. Compared to AS alone, surgery was negatively associated with concerns about treatment and subscales with items related to the physical consequences of a surgical resection, such as body image and sensations, and physical limitations in daily life or work. It has been reported that AS is associated with increased anxiety and uncertainties [27]. In the current study, patients receiving only AS did not experience greater negative physical or psychological effects than patients undergoing active treatment. Our results clearly demonstrate that the type of treatment DTF patients received, which is related to the complexity of the tumour, can have a severe impact on their HRQoL. The potential risks and benefits of treatments should therefore be considered carefully, and patients should be informed about the possible side effects associated with treatments. Since this was a cross-sectional study, it did not assess the magnitude of the impact of treatment on patients’ HRQoL over time. In future (longitudinal) studies, and clinical follow-up, the HRQoL outcome measures should be included alongside the objective outcome measures to evaluate treatment efficacy and also to facilitate shared decision making, e.g., between AS and surgery.

Differences in the time since diagnosis were only found for several subscales of the DTF-QoL and not the EORTC QLQ-C30, with significantly worse scores for patients who were ≥5 years after diagnosis. These differences were particularly seen on the impact scales, possibly reflecting the chronic character of DTF, since these items cover a timeframe since diagnosis. Another possible explanation may be that active treatments were more common in the past, and that these worse HRQoL scores are a result of these active treatments. This could explain why time since diagnosis affected only two scales after adjusting for the treatment type. A longer time since diagnosis was associated with higher scores on the supportive care subscale, indicating that these patients experienced more lack of support in the past. Therefore, these results suggest that recognition and awareness of HRQoL issues, using the DTF-QoL, is important, even long after the time of diagnosis.

Differences between tumour locations were mainly seen on the subscales of the DTF-QoL and not of the EORTC QLQ-C30, except for the pain items. These results are in line with a study of sarcoma patients by van Eck et al., who assessed HRQoL between different sarcoma locations using the EORTC QLQ-C30 and additional treatment-specific items from the EORTC Item Library [26]. They found no significant differences in the HRQoL domains of the EORTC QLQ-C30 between different tumour locations, however, they did find treatment-specific HRQoL issues that differed per sarcoma location, underlining the importance of using a disease-specific HRQoL-measurement strategy. In our study, worse scores on the DTF-specific questionnaire were observed for DTF patients with tumours in the upper and lower extremities and hip/pelvis/gluteal region on the subscales about physical limitations, pain and concerns around treatment and its consequences. These subscales, consisting of site-specific items, such as “Have you had any trouble walking?” or “Have you been afraid of needing a limb amputation?” are, therefore, particularly useful for these specific tumour sites.

The presence of comorbidities generally has a negative impact on HRQoL [28,29]. DTF patients with two or more comorbidities reported significantly worse scores on all scales and items of the EORTC QLQ-C30, which is in agreement with the previous studies conducted among patients with different types of cancer [30,31]. In addition, the results of our study indicate that the presence of comorbidities significantly affects DTF-specific HRQoL as well. Given the cross-sectional study design, it is unclear whether the self-reported comorbidities were present before a DTF diagnosis or if they developed thereafter. Moreover, comorbidities may interfere with treatment effects [29,30]. It is important to be aware of the impact of comorbidities on HRQoL, not only to assess a true treatment efficacy, but also to provide the necessary support in clinical care.

The socio-demographic factors sex, age, relationship status, education level and employment status are known to be associated with generic HRQoL [32,33,34,35]. The results of this study indicate that the female sex is not only associated with worse generic HRQoL scores, but also with DTF-specific HRQoL. It is generally assumed that HRQoL decreases with increasing age [33,36]. However, our results show that, while a higher age was negatively associated with physical symptoms, patients aged between 18 and 39 years scored significantly worse on several of the DTF-QoL impact scales. Younger DTF patients reported similar concerns to adolescent and young adult (AYA) cancer patients, e.g., concerns about their ability to have children [37,38]. The greater impact of DTF on younger patients can be explained by the fact that these patients define their identity in this period of their lives, face important life choices and often have high expectations of themselves at work and in their social lives [38]. A study by Drabbe et al. also found that AYA-sarcoma patients (aged 18–39 years) had significantly lower scores on the emotional, cognitive and social functioning scales of the EORTC QLQ-C30 compared to older patients [36]. Interestingly, in our study, a significant difference was only seen on the physical functioning scale of the EORTC QLQ-C30, with older patients scoring worse. This shows that by only using a generic questionnaire, the impact of DTF on younger patients could be missed, emphasising the importance of AYA-specific and disease-specific questionnaires [39]. It is noteworthy that, in general, socio-demographic factors had the greatest impact on generic HRQoL, whereas the influence of clinical factors was mainly seen on the DTF-QoL, indicating that the DTF-QoL provides relevant additional information about the HRQoL of these specific subgroups.

To the best of our knowledge, this is the first study exploring the heterogeneity in both the generic and disease-specific HRQoL in DTF patients. The strengths of this study are the large study population and the use of generic and disease-specific HRQoL questionnaires. Given the limited data available on HRQoL for DTF patients and the heterogeneous characteristics of DTF, the subgroup analyses are a valuable contribution to providing further insight into which patients are at risk of a poor HRQoL. Furthermore, knowledge of the differences between subgroups of DTF patients can be used to develop an individualised measurement strategy by not using all items of the DTF-QoL, but only the specific scales in which problems can be expected for that particular subgroup. For example, the parenting and fertility impact scale of the DTF-QoL could be used for patients aged 18–39 years and the physical consequences symptom scale could be used for patients with DTF located in the lower extremities or hip/pelvis/gluteal region.

The present study also has some limitations. First, there may be selection bias, as it is unknown whether DTF patients did not respond or participate, due to either the absence of symptoms or poor health [40]. The non-responder analysis did not reveal any differences, however, clinical characteristics were unavailable for these patients. Secondly, as there is no accurate national registration system in both countries, it is not possible to say with certainty which DTF patients attended the participating centres. It is assumed that at least the more complex patients were treated in the participating centres, as these were tertiary referral centres. However, it is unknown how many more complex cases have remained in the peripheral hospitals, which may also have led to selection bias. Thirdly, although we were able to analyse the clinically relevant subgroups of DTF patients, differences may also exist within these groups. Due to small numbers, we did not assess these differences in HRQoL scores. The future use of the DTF-QoL in large international cohorts will provide more data to investigate these differences within subgroups. Fourthly, the cross-sectional study design limits the possibility of drawing conclusions about causal associations. In addition, tumour behaviour was not included in our analyses. Since some DTF patients were discharged at the time of the questionnaire, the information regarding their current disease status was unavailable. Furthermore, tumour behaviour can vary during follow-up due to the unpredictable biological behaviour, making it difficult to classify patients into one particular group and to draw any conclusions about the association between the tumour’s behaviour and HRQoL. A longitudinal assessment of HRQoL data will help to determine the impact of socio-demographic and clinical factors on HRQoL over time.

## 5. Conclusions

DTF can result in a wide variety of disease-specific issues and the impact of DTF on HRQoL differs between subgroups. The use of the DTF-QoL, alongside generic HRQoL instruments, is essential to gain insight into the patient’s specific problems and needs. Together, these insights will help clinicians to provide better and more personalised care to patients with DTF.

## Figures and Tables

**Table 1:** Desmoid-type fibromatosis patient characteristics (*N* = 235).

		***n* (%)**
Nationality	United Kingdom	79 (33.6)
	The Netherlands	156 (66.4)
Sex	Male	62 (26.4)
	Female	173 (73.6)
Age in years at time of diagnosis (in years)—Mean (SD)		41.7 (14.4)
Age in years at time of questionnaire (in years)—Mean (SD)		47.2 (14.0)
Time since diagnosis (in years)—Mean (SD)		5.7 (4.5)
Tumour localization	Head/neck	13 (5.5)
	Upper extremity/shoulder	29 (12.3)
	Trunk ^1^	54 (23.0)
	Abdominal wall	58 (24.7)
	Intra-abdominal	39 (16.6)
	Hip/pelvis/gluteal region	20 (8.5)
	Lower extremity	22 (9.4)
Recurrent disease after surgery (*n* = 98, 41.7%)	Yes	41 (41.8)
	No	57 (58.2)
Treatment received ^2^	Only active surveillance	87 (37.0)
	Only surgery	64 (27.2)
	Only systemic therapy	32 (13.6)
	Only local therapy	8 (3.4)
	Combination of active treatments	44 (18.7)
Comorbidity (self-report)	None	90 (38.3)
	1	74 (31.5)
	≥2	71 (30.2)
Relationship status	Partnered	181 (77.0)
	Not partnered	53 (22.6)
	Missing	1 (0.4)
Education level	Low (primary/secondary)	36 (15.3)
	Medium (vocation/college/diploma)	126 (53.6)
	High (university/post-graduate)	73 (31.1)
Current employment status	Working	155 (66.0)
	Not working	80 (33.9)

^1^. Including thoracic wall, breast and back. ^2^. Active surveillance, surgery, systemic therapy or local therapy only: including patients who received analgesics; Systemic therapy includes: chemotherapy, hormonal therapy and targeted medical therapy (tyrosine kinase and gamma-secretase inhibitors); Local therapy includes: radiotherapy, isolated limb perfusion, high-intensity-focused ultrasound, cryoablation; Combination of active treatments: including patients who received different combinations of surgery, systemic therapy or local therapy.

**Table 2:** Mean DTF-QoL scores (±SD) in relation to socio-demographic and clinical characteristics.

	DTF-QoL													
	Symptom Scales ^+^			Impact Scales ^+^										
	W1 Emotional	W2 Physical	W3 Pain	1 Concerns Condition	2 Job & Education	3 Doctor-Patient	4 Relation-ships	5 Physical Consequences	6 Diagnostic	7 Parenting	8 Body Image	9 Support	10 Treatment Concerns	11 Behaviour DTF
Study population	15.3 (18.7)	11.6 (16.5)	19.6 (25.6)	41.3 (24.1)	29.2 (31.6)	26.7 (19.7)	24.8 (21.8)	18.7 (18.9)	28.1 (19.1)	21.1 (23.1)	29.0 (25.9)	36.0 (14.1)	22.1 (21.4)	38.8 (22.4)
Age (years)														
18–39	16.1 (19.8)	10.9 (16.4)	22.0 (28.5)	44.6 (24.4)	31.8 (31.9)	27.8 (20.7)	28.3 (22.4)	20.8 (19.6)	29.6 (20.0)	28.2 (25.4)	33.2 (26.8)	36.3 (14.6)	25.9 (23.2)	42.5 (23.9)
≥40	14.6 (17.5)	12.2 (16.7)	17.1 (22.1)	38.0 (23.5)	25.6 (30.9)	25.7 (18.6)	21.3 (20.7)	16.7 (18.1)	26.5 (18.2)	8.6 (10.1)	24.8 (24.5)	35.7 (13.7)	18.2 (18.8)	35.0 (20.3)
*p*-value	0.905	0.159	0.344	**0.024**	0.088	0.518	**0.004**	0.124	0.229	**<0.001**	**0.007**	0.943	**0.019**	**0.032**
Sex														
Male	12.4 (15.2)	7.5 (11.5)	15.1 (22.6)	39.3 (24.3)	25.4 (28.9)	22.8 (16.3)	20.5 (21.5)	14.0 (15.1)	25.3 (18.1)	8.7 (13.3)	21.5 (19.7)	34.5 (13.2)	22.4 (19.1)	37.9 (21.9)
Female	16.4 (19.7)	13.1 (17.8)	21.2 (26.4)	42.0 (24.1)	30.7 (32.5)	28.1 (20.6)	26.4 (21.8)	20.5 (19.9)	29.0 (19.4)	25.6 (24.3)	31.7 (27.4)	36.5 (14.4)	21.9 (22.2)	39.2 (22.7)
*p*-value	0.264	0.055	0.097	0.443	0.476	0.099	**0.013**	**0.044**	0.158	**<0.001**	**0.017**	0.271	0.502	0.719
Relationship status														
Partnered	14.0 (18.3)	10.6 (15.4)	19.2 (24.9)	41.0 (24.5)	27.2 (30.6)	26.8 (19.6)	23.2 (21.1)	17.0 (18.0)	28.0 (19.3)	22.1 (23.4)	26.7 (24.9)	35.9 (14.4)	21.5 (21.5)	38.7 (22.6)
Not partnered	20.0 (19.2)	15.2 (19.6)	21.0 (28.0)	42.0 (23.2)	36.5 (34.8)	26.8 (20.1)	30.2 (23.9)	24.5 (21.3)	27.7 (18.4)	15.2 (20.6)	37.6 (27.8)	36.1 (13.2)	23.7 (21.3)	39.4 (22.4)
*p*-value	**0.017**	0.122	0.779	0.755	0.095	0.914	0.050	**0.020**	0.999	0.185	**0.005**	0.551	0.430	0.837
Education level														
Low	16.1 (17.1)	14.3 (21.0)	18.5 (23.8)	34.8 (22.5)	21.1 (28.4)	25.7 (20.8)	21.2 (19.2)	16.1 (17.6)	23.5 (16.8)	15.3 (22.7)	28.8 (25.2)	33.3 (13.5)	16.9 (16.4)	36.4 (21.9)
Medium	16.0 (19.6)	11.4 (15.7)	19.5 (25.5)	43.3 (23.7)	35.2 (33.3)	27.1 (18.8)	25.2 (21.6)	20.8 (19.3)	29.5 (19.7)	19.5 (22.1)	27.8 (25.2)	35.1 (12.4)	21.7 (20.2)	39.3 (22.3)
High	13.8 (17.9)	10.7 (15.5)	20.1 (26.8)	41.0 (25.3)	21.7 (27.7)	26.6 (20.8)	25.8 (23.5)	16.5 (18.8)	27.9 (19.1)	25.0 (24.6)	31.3 (27.7)	38.8 (16.7)	25.2 (25.2)	39.3 (23.1)
*p*-value ^#^	0.712	0.554	0.956	0.181	**0.011 ^a^**	0.928	0.548	0.212	0.259	0.369	0.657	0.097	0.192	0.781
Employment status														
Working	13.6 (17.4)	9.5 (15.1)	18.4 (24.8)	41.5 (24.1)	22.9 (25.9)	26.7 (20.2)	23.4 (19.9)	16.3 (16.9)	28.5 (19.1)	20.2 (23.7)	28.0 (24.9)	36.0 (14.9)	19.8 (19.9)	37.6 (21.7)
Not working	18.6 (20.5)	15.6 (18.4)	21.9 (27.0)	40.7 (24.2)	50.5 (39.4)	26.8 (18.8)	27.6 (25.0)	23.5 (21.7)	27.1 (19.3)	24.3 (20.8)	31.0 (27.9)	36.0 (14.9)	26.5 (23.6)	41.3 (23.9)
*p*-value	0.053	**0.002**	0.307	0.752	**<0.001**	0.876	0.488	**0.017**	0.597	0.210	0.529	0.331	0.063	0.298
Comorbidity														
None	11.3 (15.8)	8.9 (14.7)	16.2 (23.7)	37.3 (22.0)	24.9 (29.4)	23.3 (21.4)	18.6 (18.7)	14.7 (17.3)	27.4 (21.4)	17.7 (21.1)	24.7 (23.2)	33.5 (13.1)	19.8 (19.7)	34.0 (20.7)
1	16.9 (19.6)	9.9 (14.6)	17.8 (26.4)	41.5 (25.0)	26.1 (30.4)	26.2 (16.8)	26.5 (21.6)	17.5 (17.3)	26.1 (17.7)	21.5 (25.1)	29.4 (27.6)	36.5 (12.1)	19.7 (21.7)	37.5 (22.3)
≥2	18.7 (20.2)	16.8 (19.3)	25.6 (26.3)	46.1 (25.2)	38.8 (34.3)	31.6 (19.4)	31.0 (23.9)	25.1 (20.9)	30.9 (17.4)	26.4 (24.7)	34.1 (26.9)	38.7 (16.7)	27.6 (22.6)	46.3 (23.0)
*p*-value ^#^	**0.028 ^b^**	**0.005 ^b,c^**	0.050	0.074	**0.026 ^b^**	**0.027 ^b^**	**0.001 ^b^**	**0.002 ^b,c^**	0.295	0.271	0.074	0.067	0.048 *	**0.002 ^b^**
Time since diagnosis														
<5 years	14.2 (17.2)	9.5 (14.3)	20.5 (26.3)	38.8 (24.3)	25.8 (30.5)	24.1 (19.0)	22.2 (21.2)	16.7 (17.1)	27.9 (18.2)	14.4 (18.2)	24.4 (23.6)	33.6 (13.1)	18.7 (18.5)	37.1 (23.2)
≥5 years	16.6 (20.2)	14.2 (18.6)	18.3 (24.7)	44.3 (23.6)	33.1 (32.4)	30.0 (20.1)	28.1 (22.3)	21.2 (20.8)	28.3 (20.2)	26.1 (25.1)	34.7 (27.6)	38.9 (14.8)	25.8 (23.7)	40.9 (21.4)
*p*-value	0.670	**0.041**	0.488	0.057	**0.040**	**0.016**	**0.021**	0.163	0.892	**0.013**	**0.003**	**0.002**	**0.043**	0.102
Treatment received ^1^														
Only active surveillance	9.1 (14.0)	6.5 (10.9)	13.9 (21.4)	31.1 (18.1)	10.3 (16.8)	25.7 (16.7)	15.7 (15.7)	8.8 (11.6)	23.5 (15.4)	17.4 (23.0)	15.6 (15.6)	34.0 (11.3)	10.3 (14.5)	29.2 (18.6)
Only surgery	12.9 (16.7)	11.1 (18.5)	15.0 (22.8)	37.2 (25.3)	28.5 (28.4)	29.0 (26.3)	22.9 (19.8)	18.6 (18.1)	27.0 (20.7)	12.0 (19.4)	28.3 (24.5)	39.1 (14.5)	22.2 (19.9)	34.7 (21.3)
Other treatment	23.5 (21.4)	17.2 (18.0)	28.8 (28.9)	55.0 (22.5)	49.2 (33.4)	26.1 (16.4)	35.8 (24.1)	29.3 (20.2)	33.6 (20.2)	32.6 (21.8)	43.4 (28.1)	35.7 (16.1)	32.8 (22.3)	51.9 (20.8)
*p*-value ^#^	**<0.001 ^d,e^**	**<0.001 ^d^**	**<0.001 ^d,e^**	**<0.001 ^d,e^**	**<0.001 ^d,e,f^**	0.563	**<0.001 ^d,e^**	**<0.001 ^d,e,f^**	**0.002 ^d^**	**<0.001 ^d,e^**	**<0.001 ^d,e,f^**	0.081	**<0.001 ^d,e,f^**	**<0.001 ^d,e^**
Recurrent disease														
Yes	18.7 (20.2)	16.7 (20.6)	20.9 (25.5)	52.7 (22.9)	44.0 (32.7)	27.6 (18.2)	29.3 (23.2)	26.0 (20.9)	29.7 (21.4)	28.7 (26.4)	39.1 (28.1)	36.3 (16.1)	34.1 (21.4)	49.3 (21.7)
No	14.6 (18.3)	10.5 (15.3)	19.3 (25.6)	38.8 (23.7)	25.9 (30.5)	26.6 (20.0)	23.9 (21.4)	17.2 (18.2)	27.7 (18.7)	19.5 (22.2)	26.9 (25.0)	35.9 (13.7)	19.3 (20.5)	36.6 (22.0)
*p*-value	0.114	0.068	0.645	**0.001**	0.001	0.623	0.098	**0.005**	0.671	0.091	**0.006**	0.671	**<0.001**	**0.001**
Recurrent disease after surgery (*n* = 98)														
Yes	18.7 (20.2)	16.7 (20.6)	20.9 (25.5)	52.7 (22.9)	44.0 (32.7)	27.6 (18.2)	29.3 (23.2)	26.0 (20.9)	29.7 (21.4)	28.7 (26.4)	39.1 (28.1)	36.3 (16.1)	34.1 (21.4)	49.3 (21.7)
No	11.9 (15.8)	9.6 (14.8)	13.4 (21.3)	35.6 (24.8)	32.1 (29.5)	29.8 (26.4)	23.2 (20.2)	19.5 (17.7)	28.7 (20.7)	12.6 (15.7)	32.0 (27.6)	39.7 (15.2)	18.4 (18.1)	32.9 (19.0)
*p*-value	**0.040**	0.063	0.162	**0.001**	0.078	0.971	0.150	0.132	0.820	**0.018**	0.190	0.362	**<0.001**	**<0.001**
Tumour location														
Abdominal wall	14.4 (17.2)	8.8 (17.4)	15.2 (24.1)	35.0 (23.8)	19.9 (28.0)	27.2 (20.2)	24.2 (23.5)	16.2 (20.9)	27.3 (19.3)	18.2 (20.9)	25.0 (26.1)	35.2 (12.0)	17.1 (20.8)	34.0 (23.6)
Intra-abdominal	11.8 (19.0)	8.0 (16.0)	8.0 (16.5)	41.7 (26.1)	28.0 (26.5)	24.2 (18.4)	20.7 (19.6)	16.6 (16.0)	23.7 (16.9)	13.5 (18.7)	16.5 (18.8)	37.9 (11.9)	15.4 (16.4)	34.5 (22.9)
Upper extremity	19.1 (17.6)	9.9 (10.9)	31.4 (25.0)	42.7 (23.1)	29.5 (32.6)	25.1 (13.3)	26.0 (23.1)	16.8 (18.3)	28.7 (20.0)	16.1 (14.4)	34.4 (24.5)	37.5 (17.2)	33.8 (23.0)	43.3 (22.0)
Lower extremity	17.8 (20.8)	25.8 (21.3)	21.2 (22.7)	42.0 (21.1)	43.5 (37.0)	24.0 (18.5)	30.0 (18.8)	29.3 (21.6)	31.8 (17.2)	31.2 (30.4)	40.3 (25.6)	40.2 (11.4)	37.8 (23.6)	45.7 (20.9)
Head/neck	18.9 (23.8)	11.3 (14.8)	22.2 (36.3)	46.2 (25.5)	60.0 (32.5)	26.9 (21.7)	27.6 (24.8)	25.0 (20.0)	31.9 (23.9)	35.6 (36.7)	37.8 (32.9)	29.1 (10.7)	20.4 (16.5)	42.7 (21.7)
Trunk	13.8 (17.7)	8.8 (11.4)	17.7 (22.2)	40.1 (23.4)	21.4 (29.3)	28.5 (22.7)	23.1 (20.8)	15.0 (15.3)	29.6 (20.6)	22.3 (28.5)	29.6 (26.7)	35.4 (17.1)	17.9 (18.7)	38.1 (21.0)
Hip/pelvis/gluteal region	18.3 (20.6)	21.3 (18.4)	38.9 (33.3)	55.8 (23.1)	40.4 (29.7)	30.8 (21.2)	30.2 (23.5)	27.2 (19.7)	26.9 (16.9)	35.4 (21.2)	37.5 (23.8)	33.9 (14.2)	25.6 (22.2)	47.1 (22.2)
*p*-value ^#^	0.613	**<0.001 ^g,h,i,j,k,l^**	**<0.001 ^h,j,m,n^**	0.062	**0.001 ^o,p^**	0.862	0.607	0.012 *	0.687	0.153	**0.003 ^i^**	0.341	**<0.001 ^g,i,l,m,q,r^**	0.109

DTF-QoL scales: W1: emotional and psychological consequences; W2: physical consequences; W3: pain and discomfort; 1: concerns about condition, 2: job and education; 3: doctor-patient relationship, communication and information; 4: effect of desmoid-type fibromatosis (DTF) on relationships; 5: physical limitations and consequences; 6: diagnostic and treatment trajectory of DTF; 7: parenting and fertility; 8: body image and sensations; 9: supportive care; 10: concerns around treatment and its consequences; 11: unpredictable course and nature of DTF. Abbreviations: conseq: consequences. ^+^ Higher scores indicate a higher level of symptomatology/problems. ^1^ Active surveillance only and surgery only: including patients who received analgesics. Other treatment, including patients who received only systemic therapy (i.e., chemotherapy, hormonal therapy, targeted medical therapy) or local therapy (i.e., radiotherapy, isolated limb perfusion, high-intensity-focused ultrasound, cryoablation) or a combination of any form of active treatments. ^#^ *p*-value of ANOVA for differences between the subgroups. Bold values indicate significant variables (*p* < 0.05). * No statistically significant differences in Bonferroni post hoc analysis. ^a, b, c, d, e, f, g, h, i, j, k, l, m, o, p, q, r^ Shows which groups are significantly different according to the Bonferroni post hoc analysis (*p* < 0.05): Medium education level versus: ^a^ high; ≥ 2 comorbidities versus: ^b^ none, ^c^ 1; Other treatment versus: ^d^ surveillance only, ^e^ surgery only; Surveillance only versus: ^f^ surgery only; Abdominal wall versus: ^g^ lower extremity, ^h^ hip/pelvis/gluteal region, ^o^ head and neck, ^q^ upper extremity; Intra-abdominal versus: ^i^ lower extremity, ^j^ hip/pelvis gluteal region, ^m^ upper extremity; Lower extremity versus: ^k^ upper extremity; Trunk versus: ^l^ lower extremity, ^n^ hip/pelvis/gluteal region, ^p^ head and neck, ^r^ upper extremity.

**Table 3:** Mean EORTC QLQ-C30 scores (±SD) in relation to socio-demographic and clinical characteristics.

	EORTC QLQ-C30														
	Functional Scales ^++^						Symptom Scales/Items ^+^								
	PF	RF	EF	CF	SF	Global QoL	Fatigue	Nausea	Pain	Dysp- Noea	Sleep	Appetite Loss	Constipation	Diarrhoea	FD
Study population	86.1 (18.6)	82.1 (27.0)	79.3 (21.3)	84.8 (21.2)	83.8 (26.0)	76.3 (19.6)	23.0 (24.9)	3.2 (8.9)	22.5 (26.5)	8.8 (19.2)	25.4 (31.6)	6.0 (16.4)	10.9 (21.3)	8.9 (21.1)	10.5 (25.3)
Age (years)															
18–39	87.9 (17.9)	81.3 (28.8)	79.0 (23.7)	86.2 (22.1)	84.3 (26.2)	77.6 (16.7)	22.7 (26.1)	3.4 (9.9)	24.3 (27.9)	4.8 (12.6)	21.6 (32.0)	5.7 (16.0)	9.7 (21.0)	7.7 (19.8)	12.0 (26.8)
≥40	84.3 (19.2)	82.9 (25.3)	79.7 (18.9)	83.3 (20.2)	83.3 (26.0)	75.0 (22.1)	23.3 (23.6)	3.0 (7.7)	20.6 (25.1)	12.7 (23.4)	29.1 (31.0)	6.2 (16.9)	12.1 (21.6)	10.2 (22.4)	9.0 (23.7)
*p*-value	**0.043**	0.924	0.552	0.087	0.603	0.717	0.511	0.969	0.318	**0.006**	**0.017**	0.857	0.292	0.332	0.305
Sex															
Male	93.4 (10.2)	90.1 (22.3)	83.3 (17.9)	86.8 (18.9)	87.4 (22.1)	80.0 (15.5)	16.5 (20.9)	2.2 (7.1)	14.5 (21.0)	4.8 (13.3)	17.2 (23.2)	2.7 (11.0)	6.5 (16.9)	9.1 (21.9)	5.4 (17.3)
Female	83.5 (20.2)	79.3 (28.1)	77.9 (22.3)	84.0 (22.0)	82.6 (27.2)	75.0 (20.8)	25.3 (25.8)	3.6 (9.4)	25.3 (27.8)	10.2 (20.8)	28.3 (33.7)	7.1 (17.8)	12.5 (22.5)	8.9 (20.9)	12.3 (27.4)
*p*-value	**<0.001**	**0.002**	0.147	0.486	0.318	0.195	**0.022**	0.224	**0.005**	0.075	0.052	**0.046**	**0.040**	0.888	0.094
Relationship status															
Partnered	87.1 (16.7)	83.7 (24.9)	80.6 (20.9)	85.6 (21.1)	85.5 (24.6)	77.1 (18.7)	22.7 (24.4)	3.3 (9.2)	22.8 (25.8)	8.5 (18.3)	25.4 (31.3)	5.3 (15.0)	10.9 (21.6)	9.2 (21.4)	8.5 (22.8)
Not partnered	82.3 (24.0)	76.4 (33.1)	74.8 (22.6)	81.4 (21.6)	78.6 (29.8)	73.6 (22.6)	23.9 (26.7)	2.8 (7.8)	21.7 (29.2)	10.1 (22.3)	25.2 (33.3)	8.2 (20.6)	11.3 (20.6)	6.9 (18.9)	17.6 (31.7)
*p*-value	0.351	0.265	0.075	0.132	0.159	0.421	0.857	0.769	0.399	0.877	0.788	0.452	0.732	0.470	0.022
Education level															
Low	81.5 (24.1)	79.6 (28.5)	80.1 (21.0)	80.6 (23.7)	83.3 (26.7)	72.2 (23.1)	25.6 (27.5)	7.4 (14.6)	22.2 (26.1)	13.0 (24.3)	26.9 (32.7)	13.9 (24.4)	13.9 (21.6)	13.0 (25.5)	13.0 (27.9)
Medium	84.6 (18.5)	79.0 (28.9)	77.4 (22.6)	83.7 (21.1)	80.2 (28.1)	73.1 (19.7)	25.2 (25.2)	2.9 (8.2)	23.8 (27.5)	9.5 (18.8)	25.7 (31.9)	5.3 (16.0)	12.2 (23.7)	10.6 (23.3)	12.4 (27.2)
High	90.6 (14.6)	88.8 (21.5)	82.3 (19.0)	88.6 (19.6)	90.4 (20.4)	83.9 (15.4)	17.8 (22.3)	1.6 (4.9)	20.3 (25.2)	5.5 (16.7)	24.2 (31.1)	3.2 (9.9)	7.3 (16.0)	4.1 (12.4)	5.9 (19.5)
*p*-value^#^	**0.018 ^a^**	**0.038 ^b^**	0.285	0.129	**0.027 ^b^**	**<0.001 ^a,b^**	0.100	**0.005 ^a,c^**	0.671	0.132	0.910	**0.004 ^a,c^**	0.200	0.052	0.178
Employment status															
Working	89.8 (14.4)	86.8 (22.8)	82.3 (19.5)	88.1 (17.5)	87.7 (21.3)	78.9 (17.6)	19.7 (22.5)	2.3 (6.3)	19.7 (24.1)	7.1 (17.8)	21.1 (29.2)	4.7 (13.9)	9.2 (19.2)	8.2 (20.9)	6.7 (22.0)
Not working	78.8 (23.2)	73.1 (32.0)	73.6 (23.7)	78.3 (25.9)	76.3 (32.1)	71.3 (22.4)	29.3 (28.0)	5.0 (12.3)	27.9 (30.2)	12.1 (21.4)	33.8 (34.6)	8.3 (20.2)	14.2 (24.7)	10.4 (21.6)	17.9 (29.5)
*p*-value	**<0.001**	**<0.001**	**0.006**	**0.004**	**0.012**	**0.016**	**0.011**	0.139	0.073	**0.027**	**0.004**	0.231	0.129	0.292	**<0.001**
Comorbidity															
None	91.5 (15.2)	86.5 (24.6)	85.0 (19.5)	90.7 (15.6)	90.2 (22.3)	84.6 (14.4)	12.8 (20.1)	0.9 (4.6)	17.0 (25.7)	3.7 (12.7)	17.0 (28.8)	2.2 (9.7)	5.9 (14.6)	3.3 (14.2)	5.9 (19.7)
1	88.3 (15.0)	84.9 (25.8)	79.4 (18.1)	84.7 (22.0)	86.9 (21.4)	76.2 (17.1)	23.3 (24.1)	4.1 (10.6)	20.5 (22.8)	5.9 (16.9)	23.9 (30.0)	5.4 (14.6)	10.4 (19.8)	6.8 (15.6)	9.5 (23.1)
≥2	76.9 (22.4)	73.7 (29.6)	72.1 (24.5)	77.2 (24.1)	72.5 (31.0)	65.8 (22.8)	35.5 (25.6)	5.2 (10.4)	31.5 (29.1)	18.3 (24.4)	37.6 (33.3)	11.3 (22.5)	17.8 (27.5)	18.3 (29.2)	17.4 (31.8)
*p*-value^#^	**<0.001 ^d,e^**	**0.006 ^d,e^**	**0.001 ^d^**	**<0.001 ^d^**	**<0.001 ^d,e^**	**<0.001 ^d,e,f^**	**<0.001 ^d,e,f^**	**0.006 ^d^**	**0.002 ^d,e^**	**<0.001 ^d,e^**	**<0.001 ^d,e^**	**0.002 ^d^**	**0.002 ^d^**	**<0.001 ^d,e^**	**0.015 ^d^**
Time since diagnosis															
<5 years	87.1 (17.7)	81.1 (27.7)	79.9 (20.4)	84.2 (21.6)	85.3 (23.4)	76.8 (20.6)	23.2 (25.3)	3.5 (9.2)	23.8 (26.1)	7.0 (17.0)	24.3 (32.7)	4.9 (13.2)	9.8 (19.3)	9.8 (22.6)	7.2 (20.4)
≥5 years	84.9 (19.8)	83.3 (26.2)	78.6 (22.5)	85.4 (20.8)	82.1 (28.9)	75.7 (18.5)	22.7 (24.4)	2.8 (8.4)	20.9 (27.1)	11.0 (21.4)	26.7 (30.3)	7.2 (19.5)	12.3 (23.6)	7.9 (19.3)	14.5 (29.8)
*p*-value	0.411	0.515	0.790	0.770	0.587	0.473	0.969	0.413	0.237	0.134	0.289	0.610	0.604	0.690	0.062
Treatment received^1^															
Only active surveillance	90.2 (16.2)	88.5 (19.2)	86.3 (18.3)	88.9 (20.0)	92.0 (17.4)	80.0 (18.4)	17.9 (21.7)	3.6 (10.9)	19.5 (22.8)	8.4 (19.2)	20.3 (28.0)	5.7 (14.6)	10.7 (21.3)	4.6 (13.6)	3.1 (12.1)
Only surgery	86.4 (19.3)	88.8 (22.6)	81.4 (20.1)	83.9 (23.0)	87.2 (21.2)	78.3 (19.8)	21.5 (24.5)	3.1 (7.9)	18.8 (27.9)	6.8 (17.0)	21.4 (31.1)	5.7 (16.3)	14.1 (25.1)	12.5 (24.8)	8.9 (23.2)
Other treatment	81.6 (19.6)	70.4 (32.8)	70.5 (22.3)	81.2 (20.5)	72.8 (32.5)	71.0 (19.8)	29.4 (27.0)	2.8 (7.2)	28.4 (28.3)	10.7 (20.8)	33.7 (34.1)	6.4 (18.3)	8.7 (18.0)	10.7 (23.8)	19.4 (33.2)
*p*-value^#^	**0.010 ^g^**	**<0.001 ^g,h^**	**<0.001 ^g,h^**	0.053	**<0.001 ^g,h^**	**0.007 ^g^**	**0.008 ^g^**	0.817	0.039 *	0.455	**0.010 ^g^**	0.964	0.321	0.047 *	**<0.001 ^g,h^**
Recurrent disease															
Yes	84.5 (19.2)	80.5 (25.5)	78.0 (22.3)	82.9 (24.3)	77.2 (29.5)	78.9 (16.9)	23.0 (24.8)	4.5 (9.9)	26.8 (29.1)	4.1 (11.0)	26.0 (30.3)	6.5 (18.6)	11.4 (21.9)	16.3 (29.0)	19.5 (31.6)
No	86.4 (18.5)	82.5 (27.4)	79.6 (21.2)	85.1 (20.5)	85.2 (25.1)	75.8 (20.2)	23.0 (24.9)	2.9 (8.7)	21.6 (26.0)	9.8 (20.4)	25.3 (32.0)	5.8 (15.9)	10.8 (21.3)	7.4 (18.8)	8.6 (23.4)
*p*-value	0.766	0.451	0.782	0.799	0.074	0.433	0.947	0.284	0.304	0.124	0.738	0.912	0.929	**0.039**	**0.003**
Recurrent disease after surgery (*n* = 98)															
Yes	84.6 (19.2)	80.5 (25.5)	78.0 (22.3)	82.9 (24.3)	77.2 (29.5)	78.9 (16.9)	23.0 (24.8)	4.5 (9.9)	26.8 (29.1)	4.1 (11.0)	26.0 (30.3)	6.5 (18.6)	11.4 (21.9)	16.3 (29.0)	19.5 (31.6)
No	87.4 (18.4)	87.1 (26.4)	80.8 (19.5)	85.4 (18.9)	87.7 (20.3)	76.3 (20.7)	22.0 (25.2)	2.3 (6.6)	16.7 (27.3)	7.6 (17.8)	22.8 (33.4)	4.7 (16.0)	12.3 (24.1)	7.6 (18.9)	7.6 (20.9)
*p*-value	0.626	0.059	0.641	0.937	0.086	0.554	0.747	0.295	**0.036**	0.420	0.387	0.404	0.954	0.128	**0.019**
Tumour location															
Abdominal wall	87.1 (20.8)	82.8 (29.8)	80.2 (23.9)	83.0 (25.5)	87.4 (25.0)	76.1 (21.4)	23.0 (28.5)	4.9 (12.9)	19.3 (25.3)	9.8 (24.2)	22.4 (33.3)	8.6 (21.2)	13.8 (27.2)	7.5 (19.8)	7.5 (20.7)
Intra-abdominal	90.9 (15.0)	91.5 (16.6)	87.0 (16.4)	89.3 (18.5)	87.2 (21.8)	78.6 (22.0)	19.4 (18.1)	3.4 (8.7)	8.1 (13.2)	9.4 (20.2)	16.2 (21.5)	5.1 (16.3)	7.7 (16.2)	17.1 (30.5)	6.0 (18.5)
Upper extremity	87.4 (15.3)	81.0 (26.6)	76.1 (21.1)	81.0 (19.3)	77.0 (28.3)	75.6 (18.2)	21.1 (25.3)	1.7 (5.2)	33.9 (30.0)	6.9 (16.4)	29.9 (31.3)	2.3 (8.6)	11.5 (20.5)	3.4 (10.3)	10.3 (23.7)
Lower extremity	80.9 (21.1)	76.5 (28.0)	76.9 (23.6)	81.8 (22.9)	76.5 (31.1)	75.0 (16.1)	25.3 (28.5)	2.3 (5.9)	31.8 (30.8)	6.1 (13.2)	34.8 (37.8)	4.5 (11.7)	10.6 (21.5)	6.1 (13.2)	22.7 (37.6)
Head/neck	83.1 (18.4)	67.9 (35.7)	75.6 (24.9)	87.2 (16.9)	83.3 (28.9)	78.8 (17.9)	24.8 (28.4)	1.3 (4.6)	28.2 (35.6)	10.3 (21.0)	35.9 (37.2)	2.6 (9.2)	10.3 (21.0)	12.8 (16.9)	17.9 (37.6)
Trunk	86.4 (17.4)	84.9 (24.1)	78.5 (19.1)	86.7 (16.9)	86.1 (22.4)	76.5 (19.2)	23.7 (21.6)	3.4 (8.2)	21.3 (22.3)	9.3 (17.6)	22.2 (29.7)	6.2 (17.2)	11.1 (19.4)	9.3 (22.8)	8.6 (23.5)
Hip/pelvis/ gluteal region	78.7 (21.9)	71.7 (31.1)	73.8 (22.3)	82.5 (26.2)	79.2 (33.3)	72.5 (18.9)	27.2 (28.3)	1.7 (5.1)	32.5 (30.8)	8.3 (14.8)	36.7 (34.0)	8.3 (14.8)	8.3 (18.3)	5.0 (16.3)	15.0 (27.5)
*p*-value^#^	0.225	0.044 *	0.250	0.632	0.372	0.949	0.934	0.629	**<0.001 ^i,j,k^**	0.985	0.091	0.650	0.895	0.141	0.157

PF: physical functioning; RF: role functioning; EF: emotional functioning; CF: cognitive functioning; SF: social functioning; Global QoL: global quality of life/health status; Sleep: sleep/insomnia; FD: financial difficulties. ^++^ Higher scores indicate better functioning; ^+^ Higher scores indicate a higher level of symptomatology/problems. ^1^ Active surveillance only and surgery only: including patients who received analgesics. Other treatment, including patients who received only systemic therapy (i.e., chemotherapy, hormonal therapy, targeted medical therapy) or local therapy (i.e., radiotherapy, isolated limb perfusion, high-intensity-focused ultrasound, cryoablation) or a combination of any form of active treatments. ^#^ *p*-value of ANOVA for differences between the subgroups. Bold values indicate significant variables (*p* < 0.05). * No statistically significant differences in Bonferroni post hoc analysis. ^a, b, c, d, e, f, g, h, i, j, k^ Shows which groups are significantly different according to the Bonferroni post hoc analysis (*p* < 0.05): High education level versus: ^a^ low, ^b^ medium; Low education level versus: ^c^ medium; ≥ 2 comorbidities versus: ^d^ none, ^e^ 1; 1 comorbidity versus: ^f^ none. Other treatment versus: ^g^ surveillance only, ^h^ surgery only; Intra-abdominal versus: ^I^ upper extremity, ^j^ lower extremity, ^k^ hip/pelvis gluteal region.

**Table 4:** Standardised betas of multiple linear regression models evaluating the association of independent variables (*p* < 0.05) with the DTF-QoL, using backward elimination.

	DTF-QoL													
	Symptom Scales ^+^			Impact Scales ^+^										
	W1 Emotional	W2 Physical	W3 Pain	1 Concerns Condition	2 Job & Education	3 Doctor-Patient	4 Relation-ships	5 Physical Consequences	6 Diagnostic	7 Parenting	8 Body Image	9 Support	10 Treatment Concerns	11 Behaviour DTF
Age	-	0.152 *	-	−0.123 *	-	-	−0.185 **	-	-	−0.324 **	−0.132 *	-	−0.137 *	−0.167 **
Sex	-	−0.138 *	-	-	−0.169 **	-	-	−0.187 **	-	−0.275 **	−0.191 **	-	-	-
Relationship status	-	-	-	-	-	-	-	-	-	-	-	-	-	-
Education level														
Low	-	-	-	-	-	-	-	-	-	-	-	Ref	-	-
Medium	-	-	-	-	-	-	-	-	-	-	-	0.057	-	-
High	-	-	-	-	-	-	-	-	-	-	-	0.215 *	-	-
Employment status	-	-	-	-	−0.260 **	-	-	-	-	-	-	-	-	-
Comorbidity														
None	Ref	Ref	Ref	Ref	-	Ref	Ref	Ref	-	-	Ref	Ref	Ref	Ref
1	0.157 *	0.030	0.086	0.133 *	-	0.069	0.227 **	0.085	-	-	0.127 *	0.117	0.080	0.133 *
≥2	0.156 *	0.190 **	0.203 **	0.167 *	-	0.195 **	0.268 **	0.201 **	-	-	0.137 *	0.193 *	0.191 **	0.264 **
Time since diagnosis	-	-	−0.146 *	-	-	-	-	-	-	-	-	0.157 *	-	-
Treatment received ^1^														
Only active surveillance	Ref	Ref	Ref	Ref	Ref	-	Ref	Ref	Ref	Ref	Ref	-	Ref	Ref
Only surgery	0.094	0.124	0.072	0.061	0.206 **	-	0.145 *	0.247 **	0.081	−0.094	0.241 **	-	0.254 **	0.050
Other treatment	0.368 **	0.297 **	0.256 **	0.415 **	0.502 **	-	0.417 **	0.529 **	0.252 **	0.335 **	0.522 **	-	0.443 **	0.414 **
Recurrence	-	-	-	0.138 *	0.141 *	-	-	-	-	-	-	-	-	0.136 *
Tumour location														
Abdominal wall	-	Ref	Ref	-	-	-	-	-	-	-	-	-	Ref	-
Intra-abdominal	-	−0.068	−0.148 *	-	-	-	-	-	-	-	-	-	−0.099	-
Upper extremity	-	−0.054	0.131	-	-	-	-	-	-	-	-	-	0.144 *	-
Lower extremity	-	0.259 **	0.037	-	-	-	-	-	-	-	-	-	0.198 **	-
Head/neck	-	−0.048	−0.011	-	-	-	-	-	-	-	-	-	−0.056	-
Trunk	-	−0.065	−0.026	-	-	-	-	-	-	-	-	-	−0.022	-
Hip/pelvis/ gluteal region	-	0.190 **	0.223 **	-	-	-	-	-	-	-	-	-	0.023	-

Age: 18–39 vs. ≥40 years; sex: female vs. male; relationship status: non-partnered vs. partnered; current employment status: not working vs. working; comorbidity: none vs. 1; none vs. ≥2; time since diagnosis: <5 years vs. ≥5 years; recurrence: no recurrence vs. recurrence. DTF-QoL scales: W1: emotional and psychological consequences; W2: physical consequences; W3: pain and discomfort; 1: concerns about condition; 2: job and education; 3: doctor-patient relationship, communication and information; 4: effect of desmoid-type fibromatosis (DTF) on relationships; 5: physical limitations and consequences; 6: diagnostic and treatment trajectory of DTF; 7: parenting and fertility; 8: body image and sensations; 9: supportive care; 10: concerns around treatment and its consequences; 11: unpredictable course and nature of DTF. ^1^ Active surveillance only and surgery only: including patients who received analgesics. Other treatment, including patients who received only systemic therapy (i.e., chemotherapy, hormonal therapy, targeted medical therapy) or local therapy (i.e., radiotherapy, isolated limb perfusion, high-intensity-focused ultrasound, cryoablation) or a combination of any form of active treatments. * *p* < 0.05; ** *p* < 0.01. ^+^ Higher scores indicate a higher level of symptomatology/problems.

## Data Availability

The datasets used and/or analysed during the current study are available from the corresponding author on reasonable request.

## Supplementary Materials

The following are available online at https://www.mdpi.com/article/10.3390/cancers14122979/s1, Table S1: Specification of active treatment types (*n* = 148); Table S2: Mean DTF-QoL single item scores (±SD) in relation to socio-demographic and clinical characteristics.
