# Supplementary material for: Unraveling Desmoid-Type Fibromatosis-Specific Health-Related Quality of Life: Who Is at Risk for Poor Outcomes
Source: Cancers (Basel). 2022 Jun 16;14(12):2979. doi: 10.3390/cancers14122979 (PMC9221474; doi:10.3390/cancers14122979)
Supplement: Supplementary file 1 [file cancers-14-02979-s001.zip › Supplementary Table S1.pdf]

Table S1 Specification of active treatment types (n=148)

		n (%)*
Surgery		98 (66.2)
Systemic therapy	Total	60 (40.5)
	Chemotherapy [a]	37 (25.0)
	Hormonal therapy [b]	28 (18.9)
	Targeted medical therapy [c]	15 (10.1)
Local therapy	Total	43 (29.1)
	Radiotherapy	34 (23.0)
	Isolated limb perfusion	5 (3.4)
	High-intensity focused ultrasound	2 (1.4)
	Cryoablation	6 (4.1)

* Percentage of the total number of patients with desmoid-type fibromatosis who received an active treatment. Patients could have received multiple types of active treatments

[a] n=23 vinorelbine, n=4 vinblastine/methotrexate, n=14 doxorubicin, n=1 VAC (vincristine, actomycin-D, cyclophosphamide)

[b] Hormonal therapy includes: tamoxifen

[c] Targeted medical therapy include: tyrosine kinase and gamma-secretase inhibitors
