# Supplementary material for: Unraveling Desmoid-Type Fibromatosis-Specific Health-Related Quality of Life: Who Is at Risk for Poor Outcomes
Source: Cancers (Basel). 2022 Jun 16;14(12):2979. doi: 10.3390/cancers14122979 (PMC9221474; doi:10.3390/cancers14122979)
Supplement: Supplementary file 1 [file cancers-14-02979-s001.zip › Supplementary Table S2.pdf]

Table S2. Mean DTF-QoL single item scores (±SD) in relation to socio-demographic and clinical characteristics

	DTF-QoL single items [+]					
	Decreased libido	Hair loss	DTF changed life in negative way	Hair colour change	Wasting time of cancer specialists	Rash treatment
Age (years)						
18-39	23.1 (31.4)	11.7 (25.6)	39.3 (34.4)	6.3 (19.8)	14.2 (26.0)	14.0 (28.4)
≥40	28.1 (36.3)	10.2 (23.3)	27.7 (31.2)	7.4 (18.8)	9.0 (19.3)	10.1 (25.0)
P-value	0.442	0.703	**0.007**	0.364	0.136	0.174
Sex						
Male	16.4 (29.6)	5.4 (18.3)	30.6 (31.5)	8.8 (24.8)	4.3 (11.3)	12.9 (26.6)
Female	28.9 (34.7)	12.9 (26.1)	34.5 (33.9)	6.1 (16.8)	14.3 (25.5)	11.7 (26.9)
P-value	**0.011**	**0.016**	0.499	0.857	**0.006**	0.527
Relationship status						
Partnered	25.9 (33.9)	11.4 (25.1)	32.2 (32.0)	7.0 (19.4)	11.0 (21.7)	10.6 (25.6)
Not partnered	23.8 (33.9)	8.8 (21.8)	37.7 (37.6)	6.4 (19.2)	13.8 (27.3)	16.4 (30.4)
P-value	0.714	0.512	0.469	0.815	0.730	0.122
Education level						
Low	24.0 (35.4)	4.6 (14.1)	25.9 (28.9)	6.7 (22.1)	12.0 (22.8)	6.5 (15.6)
Medium	26.8 (32.8)	12.4 (26.6)	36.5 (35.4)	7.1 (19.7)	8.5 (18.8)	12.1 (27.3)
High	23.8 (35.0)	11.4 (24.4)	32.0 (31.1)	6.3 (17.4)	16.9 (28.4)	14.6 (29.9)
P-value[#]	0.826	0.236	0.218	0.958	**0.044**[a]	0.330
Employment status						
Working	23.1 (31.4)	8.6 (21.1)	31.8 (31.2)	4.8 (16.1)	12.7 (24.1)	11.7 (26.8)
Not working	30.7 (38.5)	15.4 (29.5)	36.7 (37.0)	11.5 (24.6)	9.6 (20.7)	12.7 (26.8)
P-value	0.299	0.133	0.516	**0.021**	0.328	0.662
Comorbidity						
None	16.5 (30.1)	7.4 (22.2)	31.1 (31.9)	5.1 (17.5)	12.6 (23.7)	12.2 (26.7)
1	25.8 (35.0)	12.6 (25.1)	32.0 (33.8)	4.6 (16.5)	8.6 (19.2)	6.9 (21.6)
≥2	37.9 (33.9)	13.6 (26.2)	38.0 (34.4)	11.5 (23.5)	13.6 (25.6)	16.9 (30.8)
P-value[#]	**0.001**[b]	0.215	0.382	0.078	0.368	0.084
Time since diagnosis						
<5 years	25.9 (34.4)	10.3 (25.6)	32.8 (32.8)	4.4 (14.4)	11.1 (21.8)	8.9 (24.6)
≥5 years	24.9 (33.2)	11.6 (23.0)	34.3 (33.9)	9.5 (23.4)	12.3 (24.5)	15.7 (28.8)
P-value	0.928	0.229	0.787	0.068	0.875	**0.008**
Treatments received[1]						
Only surveillance	21.6 (30.0)	6.9 (19.8)	21.8 (26.3)	2.6 (10.5)	9.6 (19.6)	0.8 (5.1)
Only surgery	18.6 (29.9)	5.2 (14.8)	25.5 (30.1)	5.6 (16.8)	12.0 (24.0)	6.9 (19.1)
Other	35.2 (38.6)	19.4 (31.6)	51.6 (34.5)	11.7 (25.5)	13.5 (25.4)	27.4 (36.3)
P-value[#]	**0.009**[c,d]	**<0.001**[c,d]	**<0.001**[c,d]	**0.011**[c]	0.535	**<0.001**[c,d]
Recurrent disease						
Yes	23.1 (30.7)	8.1 (19.4)	46.3 (32.3)	7.9 (25.0)	13.0 (26.7)	23.6 (36.7)
No	26.0 (34.5)	11.5 (25.4)	30.8 (32.9)	6.6 (17.8)	11.3 (22.2)	9.5 (23.5)
P-value	0.734	0.529	**0.003**	0.618	0.991	**0.005**
Recurrent disease after surgery (n=98)						
Yes	23.1 (30.7)	8.1 (19.4)	46.3 (32.4)	7.9 (25.0)	13.0 (26.7)	23.6 (36.7)
No	20.9 (32.6)	8.8 (23.2)	26.3 (33.2)	6.1 (17.6)	10.5 (22.9)	8.3 (23.1)
P-value	0.625	0.897	**0.002**	0.689	0.685	**0.010**
Tumour location						
Abdominal wall	24.7 (35.0)	8.6 (20.3)	27.0 (34.5)	3.3 (10.1)	12.6 (22.4)	4.2 (14.3)
Intra-abdominal	17.2 (32.9)	14.5 (32.3)	29.9 (32.3)	10.8 (22.8)	5.1 (14.4)	11.1 (24.6)
Upper extremity	33.3 (39.5)	4.6 (14.7)	34.5 (28.8)	14.3 (30.7)	12.6 (16.5)	20.7 (36.1)
Lower extremity	20.0 (27.4)	10.6 (21.5)	48.5 (35.2)	13.3 (27.4)	10.6 (21.5)	25.8 (35.5)
Head/neck	26.7 (37.8)	38.5 (42.7)	38.5 (42.7)	5.6 (13.0)	5.1 (12.5)	7.7 (20.0)
Trunk	27.1 (32.0)	7.4 (16.7)	30.2 (29.2)	1.4 (9.5)	15.4 (30.2)	11.1 (27.5)
Hip/pelvis/gluteal region	29.6 (32.1)	11.7 (24.8)	46.7 (34.9)	5.6 (17.1)	15.0 (29.6)	13.3 (27.4)
P-value[#]	0.656	**0.001**[e,f,g,hi,j]	0.083	**0.032***	0.396	**0.026**[k]

[+] Higher scores indicate a higher level of symptomatology / problems.

[1] Active surveillance only and surgery only: including patients who received analgesics; Other treatment including patients who received only systemic therapy (i.e. chemotherapy, hormonal therapy, targeted medical therapy) or targeted therapy (i.e. radiotherapy, isolated limb perfusion, high-intensity focused ultrasound, cryoablation) or a combination of any form of active treatments.
Bold values indicate significant variables ($P<0.05$)
[#] *P*-value of ANOVA for differences between the subgroups
[*] No statistically significant differences in Bonferroni post hoc analysis
[a, b, c, d, e, f, g, h, i, j, k] Shows which groups are significantly different according to the Bonferroni post hoc analysis ($P<0.05$):

- Medium education level versus: [a] high
- ≥2 comorbidities versus: [b] none
- Other treatment versus: [c] surveillance only, [d] surgery only
- Head and neck versus: [e] abdominal wall, [f] intra-abdominal, [g] upper extremity, [h] lower extremity, [i] trunk, [j] hip/pelvis/gluteal region
- Abdominal wall versus: [k] lower extremity
